# Japanese Traditional Herbal Medicine, Rikkunshito, Partially Suppresses Inflammatory Responses in Myocardial Ischemia/Reperfusion Injury

**DOI:** 10.7759/cureus.54485

**Published:** 2024-02-19

**Authors:** Tomoe Sato, Yasuaki Sawashita, Yusuke Yoshikawa, Michiaki Yamakage

**Affiliations:** 1 Anesthesiology, School of Medicine, Sapporo Medical University, Sapporo, JPN; 2 Anesthesia, Sapporo Central Hospital, Sapporo, JPN

**Keywords:** cardiology, inflammation, ischemia-reperfusion injury, rikkunshito, traditional herbal medicine

## Abstract

Introduction: Myocardial ischemia/reperfusion (I/R) injury can cause additional damage to an ischemic myocardium, even after successful reperfusion therapy. Inflammation is a mechanism that exacerbates myocardial damage after I/R injury. Rikkunshito* *(RKT) is a traditional Japanese herbal medicine widely used to treat gastrointestinal symptoms. It attenuates inflammation and fibrosis in some diseases of the heart; however, it remains unclear whether RKT exerts cardioprotective effects against myocardial I/R injury. To elucidate this, we evaluated the effects of RKT pre-treatment by oral administration on the myocardium in a mouse model of in vivo I/R injury.

Methods: Mice were randomly assigned to a group receiving distilled water (DW) or one receiving RKT (1000 mg/kg/day) for 14 days orally. For each of the RKT and DW groups, a sham group, an I/R 2 h group, and an I/R 24 h group were created. On day 15, myocardial I/R surgery was performed. The left anterior descending coronary artery (LAD) was ligated for 30 min, and reperfusion time was set at 2 h or 24 h. The myocardial infarct size (IS) was measured after 2 h of reperfusion, and cardiac cytokine mRNA expression levels were evaluated by quantitative reverse transcription polymerase chain reaction (RT-PCR) after 2 h and 24 h of reperfusion.

Results: RKT pre-treatment significantly suppressed the cardiac mRNA expression level of interleukin-1β in the RKT-I/R 2 h group compared to the DW-I/R 2 h group (P < 0.05). Additionally, RKT significantly suppressed the mRNA expression levels of transforming growth factor-β compared to DW; the same result was obtained for the expression levels of interleukin-6. However, RKT did not reduce the IS or mRNA expression levels of the cardiac congestive markers natriuretic peptide a (NPPA) and natriuretic peptide b (NPPB). In addition, RKT did not alter the plasma concentration of ghrelin and sirtuin 1 (Sirt1), which have been reported to be stimulated by RKT.

Conclusion: This study showed that pre-treatment of RKT for myocardial I/R injury partially suppressed inflammation-related cytokines. However, further studies are needed on the effect of RKT on the reduction of myocardial infarction size.

## Introduction

Ischemic heart disease remains the leading cause of morbidity and mortality worldwide [1]. Rapid and successful reperfusion therapies, such as percutaneous coronary intervention or coronary artery bypass grafting, are necessary to save an ischemic myocardium; however, reperfusion itself can exacerbate myocardial damage and increase infarct size (IS) [2-5]. This phenomenon is known as myocardial ischemia-reperfusion (I/R) injury; however, its mechanisms have not been fully elucidated. Previous studies have indicated that inflammatory responses are one of the mechanisms underlying I/R injury [2,4-7]. Moreover, “acute” myocardial infarction activates an immune response [8-10]. Both innate and acquired immunities are induced, and neutrophils and monocytes are activated and mobilized, resulting in the release of cytokines, chemokines, and adhesion factors [11]. In particular, cytokines such as tumor necrosis factor-α (TNF-α), interleukin-1β (IL-1β), and IL-6 play a central role in I/R injury and propagate the inflammatory response [7,10,12]. Several animal studies support the importance of these cytokines in I/R injury; for example, anti-inflammatory agents improve left ventricular remodeling and reduce cardiomyocyte death in a mouse model of acute myocardial infarction [13,14].

Rikkunshito (RKT) is a traditional Japanese herbal medicine that is commonly used to treat gastroesophageal reflux diseases after abdominal surgery and anorexia under stressful conditions [15-21]. Recently, RKT has been reported to exhibit anti-inflammatory effects in several animal studies [18,22-25]. Oral administration of RKT suppressed lung fibrosis and inflammation-related cytokines in a mouse model of bleomycin-induced lung injury [18]. Furthermore, in a mouse model of angiotensin II-induced atrial fibrillation, RKT reduced myocardial fibrosis and inflammatory cytokine levels [24]. However, no studies have examined whether pre-treatment with RKT can prevent myocardial I/R injury by suppressing the inflammatory response. Notably, this herb is an orally administered medicine that can be easily prescribed prophylactically in clinical settings. In this study, we investigated whether the “prophylactic” administration of RKT suppresses the inflammatory response and attenuates myocardial injury in a mouse model of acute myocardial I/R injury.

## Materials and methods

Ethics statement

The study protocol was approved by the Institutional Animal Care and Use Committee of Sapporo Medical University (Protocol Number: 21-087). The study was conducted in strict accordance with the basic guidelines for conducting animal experiments at research institutes (published by the Ministry of Education, Culture, Sports, Science and Technology). All surgeries were performed under medetomidine-midazolam-butorphanol anesthesia, and all efforts were made to minimize suffering.

Formula of RKT

Dry powdered extracts of RKT (lot no. 2190043010) were supplied by Tsumura &Co. (Tokyo, Japan) and dissolved in distilled water (DW). RKT is formulated from eight kinds of herbal components [26]. The detailed components of RKT are shown in Table 1.

**Table 1:** Components of RKT 7.5 g of RKT contains 4.0 g of dried extract of the above mixed crude drugs. RKT, rikkunshito

Component herbs	Grams
Atractylodes rhizome (Atractylodis lanceae rhizoma)	4.0
Ginseng (Ginseng radix)	4.0
Pinellia tuber (Pinellia ternata)	4.0
Poria sclerotium (Poria cocos)	4.0
Jujube (Zizyphi fructus)	2.0
Citrus unshiu peel (Aurantii nobilis pericarpium)	2.0
Glycyrrhiza (Glycyrrhizae radix)	1.0
Ginger (Zingiberis rhizoma)	0.5

Animals

Male C57BL/6 mice aged eight to 12 weeks were purchased from Sankyo Co. Ltd. (Tokyo, Japan) and housed in a temperature-controlled room with a 12 h light/dark cycle (8:00 AM-8:00 PM). The mice were fed standard laboratory chow (CRF-1; Charles River Formula, Tokyo, Japan) and water ad libitum. Mice were randomly assigned to a group receiving DW or a group receiving RKT (1000 mg/kg/day) for 14 days. For each of the RKT and DW groups, a sham 2 h group, an I/R 2 h group, a sham 24 h group, and an I/R 24 h group were created. A total of 8 groups were created in this study, 4 groups treated with RKT, and 4 groups treated with DW. Because 10 mice were allocated to each group, the total number of RKT-treated mice was 40 (10 multiplied by 4), and the total number of DW-treated mice was also 40. As described above, 10 mice were allocated to each group, but there were some discrepancies in the number of samples shown in the results of each experiment due to sample processing errors or the limit of the number of samples that can be processed in each experimental procedure. The number of mice in each experimental group is reported in the respective figure legends.

RKT administration

The dose of RKT was set at 1000 mg/kg according to previous studies that showed an effective anti-inflammatory effect of RKT [18,22]. The human equivalent dose of RKT at 1000 mg/kg in mice was calculated to be 81 mg/kg according to the formula of a previous study, which is 4.86 g for a human weighing 60 kg [27]. The normal oral dose of RKT in humans is 7.5 g/day; thus, the oral dose administered to the mice in this study was lower than the normal human dose. The dose of RKT that could cause adverse effects has been reported to be greater than 2000 mg/kg according to the manufacturer’s instructions; therefore, the dose used in this study was considered to be within the recommended dose [28]. Because RKT is a dry powder, it was dissolved in DW to a concentration of 0.1 g/mL, and 0.01 ml/kg was administered. Equal amounts of DW were administered to the DW group. To ensure that the mice ingested RKT, we chose gavage rather than mixing food with RKT. Stainless steel oral feeding tubes (Instech Laboratories, Inc., PA, USA) and a 1 mL syringe (TERUMO, Tokyo, Japan) were used for gavage and were used separately for the DW and RKT groups. Body weight was measured daily before administration of DW or RKT.

Myocardial I/R injury

**Figure 1:**
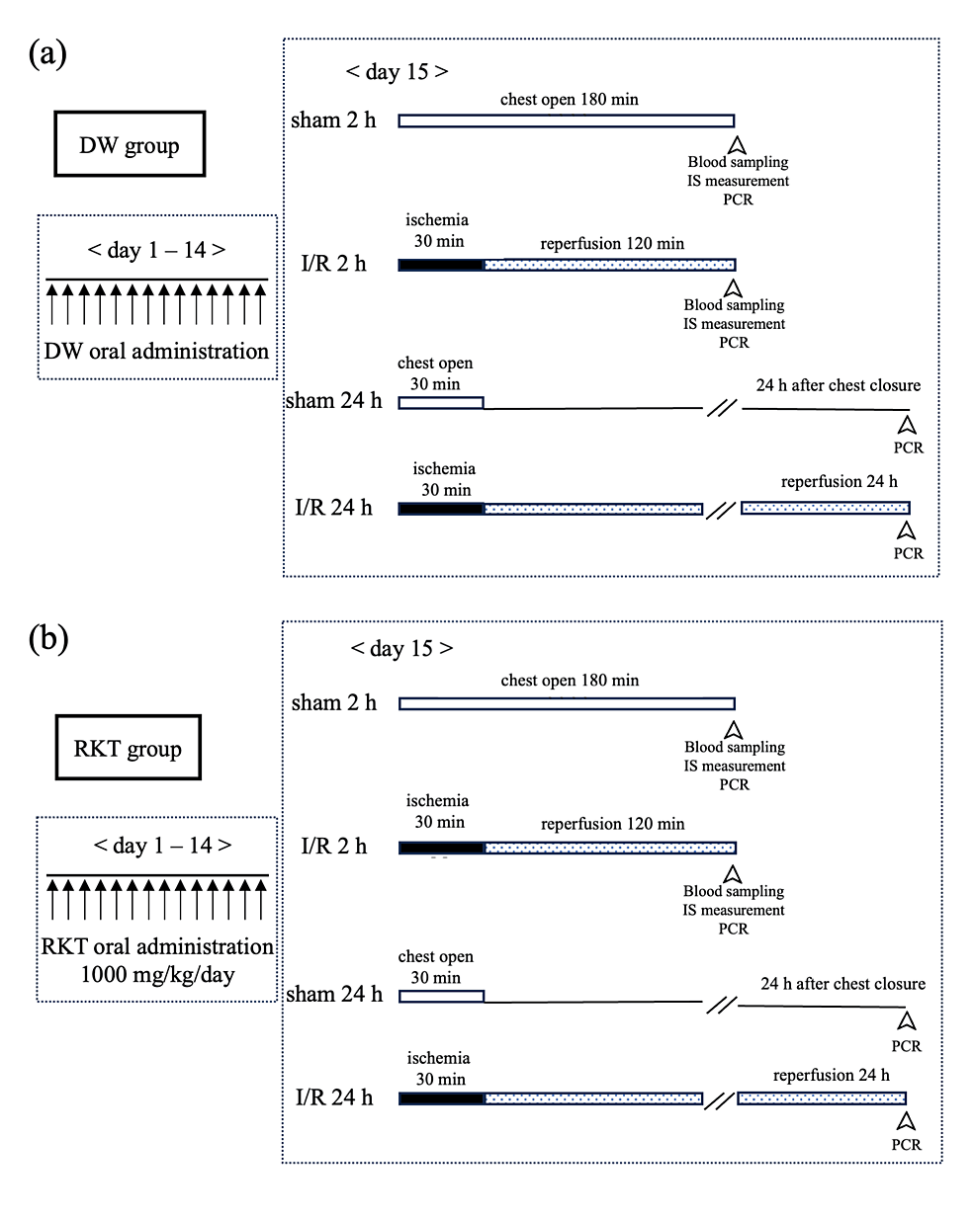
Time schedule of oral administration, I/R surgery, and timing of sample acquisition for in vivo experiments (a) In the DW group, mice received DW orally for 14 days and were operated on day 15. Mice were assigned to the following groups: DW-sham 2 h, DW-I/R 2 h, DW-sham 24 h, DW-I/R 24 h. n = 10 for each group. (b) In the RKT group, mice received RKT orally for 14 days and were operated on day 15. Mice were assigned to the following groups: RKT-sham 2 h, RKT-I/R 2 h, RKT-sham 24 h, RKT-I/R 24 h. In the sham 24 h and I/R 24 h groups for both DW and RKT, mice were awakened from anesthesia after 30 min of treatment and reanesthetized 24 h later. n = 10 for each group. RKT, rikkunshito; DW, distilled water; I/R, ischemia reperfusion; IS, infarction size; PCR, polymerase chain reaction

Myocardial I/R injury was induced by left anterior descending coronary artery (LAD) ligation according to a previously described method [29,30]. Mice were positioned on a heating pad to maintain their body temperature at 37℃. Briefly, mice were injected intraperitoneally (i.p.) with a mixture of medetomidine (0.3 mg/kg), midazolam (4 mg/kg), and butorphanol (5 mg/kg). After endotracheal intubation, the mice were ventilated in volume-controlled mode with a small animal ventilator (Model 683, Harvard Apparatus, Holliston, MA, USA). Anesthetics were added as needed if there were signs of arousal during surgery. The myocardial I/R model was established as follows. The LAD was ligated using 8-0 PROLENE^TM^ (Ethicon, NJ, USA) over a 1 mm wide piece of PE No. 10TM (Becton, Dickinson and Company, NJ, USA) just below the left atrial appendage. The heart was then subjected to 30 min of ischemia and 2 h or 24 h of reperfusion. Successful ischemia was confirmed when the heart turned pale, and reperfusion was confirmed when the myocardium recovered its blood flow and turned red. In the 2 h reperfusion group, blood sampling and heart extraction were performed at the end of reperfusion. In the 24 h reperfusion group, after 2 h of reperfusion, the chest was closed using 6-0 VICRYLTM (Ethicon), and buprenorphine 0.05 mg/kg was injected subcutaneously for postoperative pain relief before recovery from anesthesia. After awakening from anesthesia, the mice were extubated, and 24 h later, the heart was extracted under the same anesthesia as described above. In the sham group, the chest was opened, and the pericardium was incised. The timeline of all the experimental schedules is shown in Figure 1.

Determination of IS

After reperfusion for 2 h, the LAD was re-occluded, and 4% Evans Blue was injected according to previous studies [27,28]. The hearts were excised, and the left ventricles (LV) were cut into slices about 1.5 mm thick. The heart slices were incubated in a 1% solution of 2,3,5-triphenyl tetrazolium chloride for 15 min at 37℃. The percentage area at risk (AAR) and IS were evaluated using ImageJ software (National Institutes of Health, Bethesda, MD, USA) in a blinded manner. The AAR/LV and IS/LV ratios are expressed as percentages.

Measurement of ghrelin concentration

The plasma concentration of total ghrelin was determined using an enzyme-linked immunosorbent assay (ELISA) kit (LSI Medicine, Tokyo, Japan), according to the manufacturer’s instructions. Data were evaluated spectrophotometrically using a standard 96-well plate reader at a 450 nm wavelength (SunriseTM reader; Tecan Group Ltd., Männedorf, Switzerland).

Confirmation of the gene expression profiles using RT-PCR

Reverse transcription polymerase chain reaction (RT-PCR) analysis was performed according to a previous study, with some modifications [31,32]. Total RNA was extracted from the infarct area of the LV after 24 h of reperfusion using the QIAzol lysis reagent (Qiagen, Hilden, Germany). The mRNA concentration in the sample was measured using NanoDrop^TM^ (Thermo Fisher Scientific, Waltham, MA, USA). Single-stranded cDNA was retrieved using Superscript^TM^ III Reverse Transcriptase (Thermo Fisher Scientific). RT-PCR was performed using Fast SYBR^TM^ Green Master Mix and StepOnePlus^TM^ (Thermo Fisher Scientific), following the manufacturer’s protocol. Gene-specific primers for RT-PCR were designed using PrimerBLAST. The forward and reverse primers used in this study are shown in Table 2.

**Table 2:** Primer sequences used in RT-PCR RT-PCR, reverse transcriptase polymerase chain reaction; Sirt1, Sirtuin 1; NPPA, natriuretic peptide a; NPPB, natriuretic peptide b; TNFα, tumor necrosis factor-α; IL-1β, interleukin-1β; IL-6, interleukin-6; TGF-β, transforming growth factor-β; GAPDH, glyceraldehyde-3-phosphate dehydrogenase

Primer name	Direction	Sequence (5’-3’)
Sirt1	Forward	GACCTCCCAGACCCTCAAGC
	Reverse	AGAGACGGCTGGAACTGTCC
NPPA	Forward	GCTTCGGGGGTAGGATTGACA
	Reverse	GCTCAAGCAGAATCGACTGCC
NPPB	Forward	TTTGGGCACAAGATAGACCGGA
	Reverse	CCAGGCAGAGTCAGAAACTGGA
IL-1β	Forward	TGCCACCTTTTGACAGTGATGAG
	Reverse	TGCCTGCCTGAAGCTCTTGT
IL-6	Forward	ATCGTGGAAATGAGAAAAGAGTTGTG
	Reverse	ATATCCAGTTTGGTAGCATCCATCAT
TGF-β	Forward	TGCTGACCCCCACTGATACG
	Reverse	TGGGGCTGATCCCGTTGAT
GAPDH	Forward	AGGTCGGTGTGAACGGATTTG
	Reverse	TGTAGACCATGTAGTTGAGGT

Statistical analysis

Data are expressed as mean ± standard error of the mean or absolute numbers (%). The normality of the data distribution was tested using the Shapiro-Wilk test. Differences between the two independent groups were analyzed using unpaired t-tests. Body weight changes were analyzed using a two-way repeated-measures analysis of variance (ANOVA). RT-PCR data were evaluated for changes in gene expression in the heart using two-way ANOVA, comparing differences in feeding (DW, RKT), and surgery (sham, I/R). The Sidak test was performed for multiple comparisons, followed by two-way ANOVA, as required. All statistical analyses were performed using the GraphPad Prism 8 software (GraphPad Software, La Jolla, CA, USA). P less than 0.05 indicated significance. 

## Results

Body weight changes during administration of RKT

**Figure 2:**
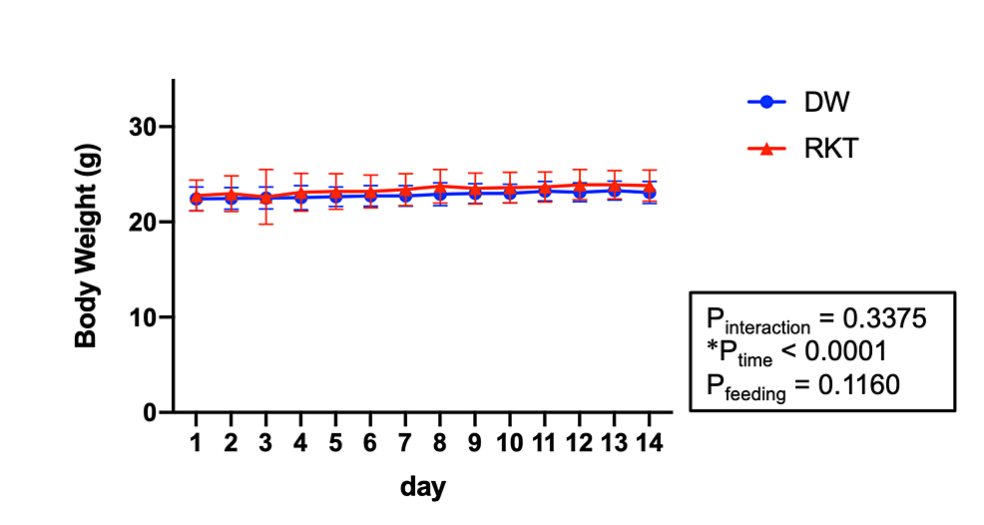
Body weight change during 14 days of oral administration of RKT or DW Data were analyzed by two-way repeated measures ANOVA. *P > 0.05; n = 40 for each group. Data are shown as means ± SEM. RKT, rikkunshito; DW, distilled water; ANOVA, analysis of variance; SEM, standard error of mean

Because RKT has been reported to have an orexigenic effect, we examined the effect of RKT on body weight [33,34]. Both groups showed significant weight gain over the 14 days (P_time _< 0.0001), but RKT had no significant effect on weight gain (P_interaction_ = 0.3375, P_feeding_ = 0.1160) (Figure 2). 

Effect of RKT on myocardial damage caused by I/R injury

**Figure 3:**
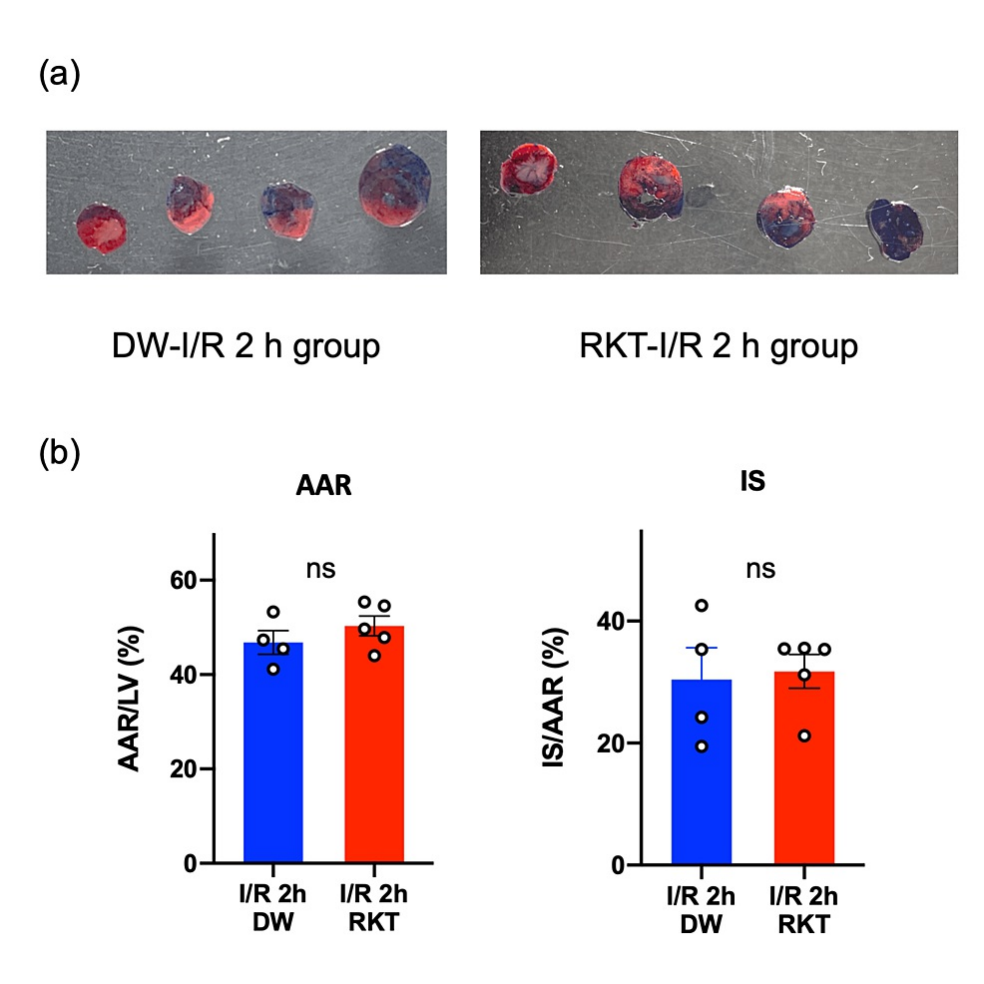
Effect of RKT and DW administration on IS (a) Representative pictures of tissue cross-sections in the DW-I/R 2h group and RKT-I/R 2h group. (b) The comparison of AAR/LV and IS/AAR between the DW-I/R 2h group and RKT-I/R 2h group. The Y-axis is expressed as a percentage. n = 4-5 in each group. Data are shown as individual plots and mean ± SEM. Data were analyzed using Student’s t-tests. ○: individual plot, ns: nonsignificant at P > 0.05. RKT, rikkunshito; DW, distilled water; AAR, area at risk; LV, left ventricle; IS, infarct size; I/R, ischemia/reperfusion; SEM, standard error of mean

**Figure 4:**
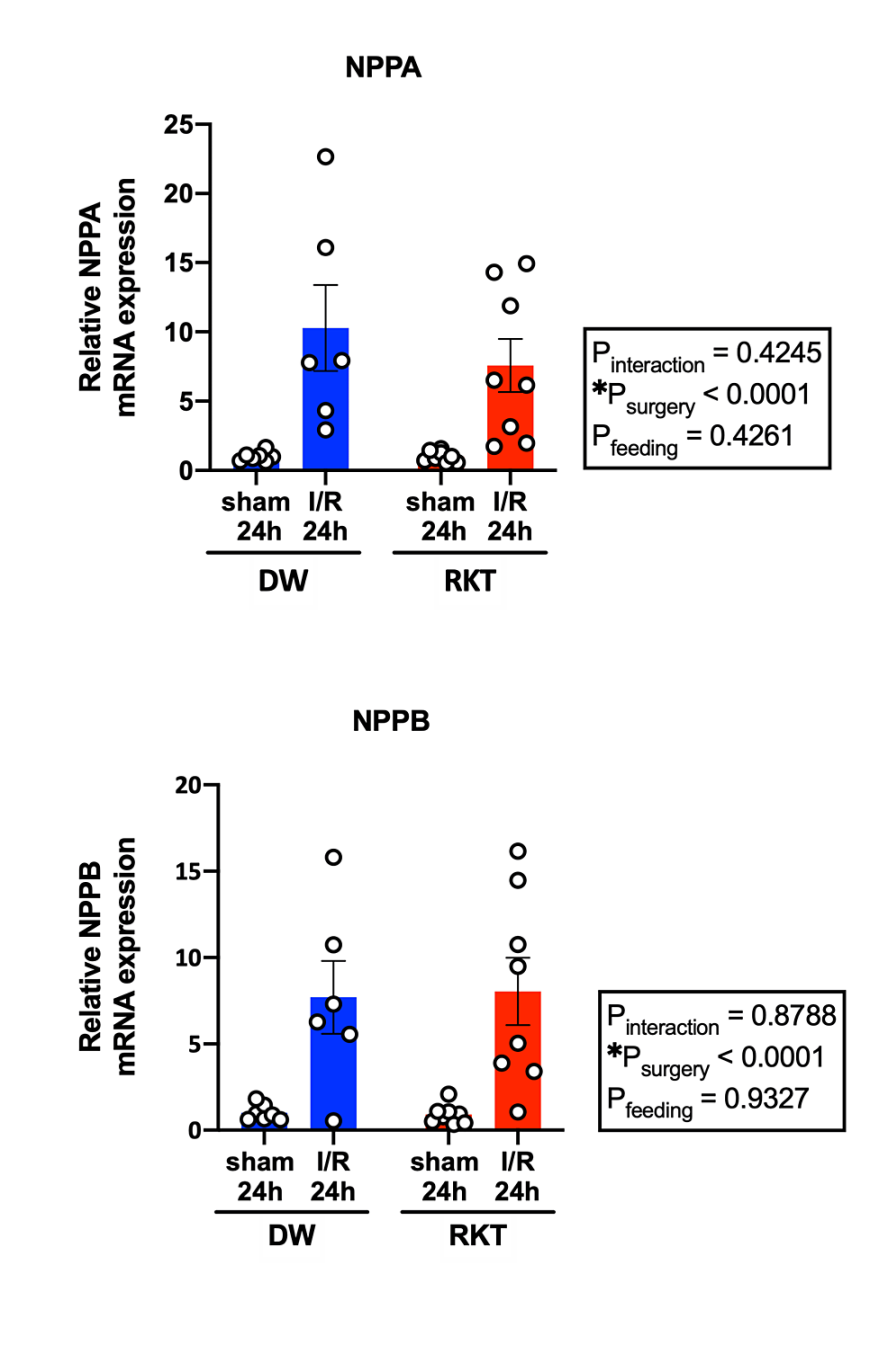
Myocardial mRNA expression levels of cardiac stress markers after 24 h of reperfusion For both NPPA and NPPB, surgery had a significant effect on upregulation of mRNA expression in both the DW-I/R 24 h and RKT-I/R 24 h groups compared to the DW-sham 24 h and RKT-sham 24 h group. n = 6-8 in each group. Data are shown as individual plots and means ± SEM. ○: individual plot, *P < 0.005. NPPA, natriuretic peptide a; NPPB, natriuretic peptide b; RKT, rikkunshito; DW, distilled water; I/R, ischemia-reperfusion; SEM, standard error of mean

To investigate the effect of RKT on I/R-induced myocardial damage, myocardial I/R injury surgery was performed in vivo, and AAR and IS were measured in the RKT-I/R 2 h and DW-I/R 2 h groups. The size of the AAR did not differ between the two groups (P = 0.3195), but RKT administration did not reduce IS (P = 0.8158) (Figure 3). mRNA expression levels of the cardiac stress markers natriuretic peptide a (NPPA) and natriuretic peptide b (NPPB) were measured to examine the degree of cardiac stress after 24 h of reperfusion (Figure 4). Both mRNA levels were upregulated after I/R surgery (both: P_surgery_ < 0.0001); however, there was no significant effect of feeding between the DW-I/R 24 h and RKT-I/R 24 h groups (NPPA: P_feeding_ = 0.4261, NPPB: P_feeding_ = 0.9327).

Effect of RKT on cardiac inflammatory response after I/R injury

**Figure 5:**
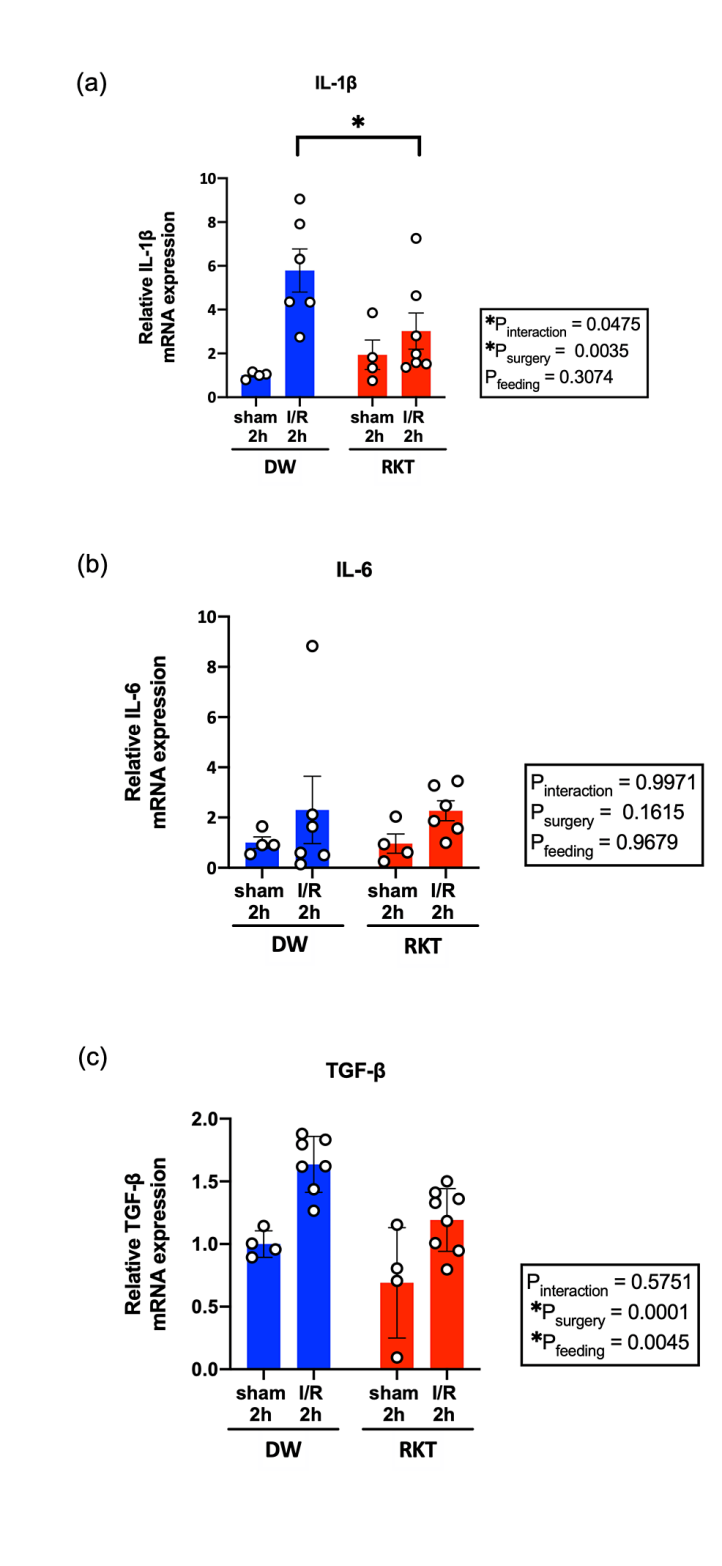
Myocardial mRNA expression levels of inflammation-related cytokines at 2 h of reperfusion (a) relative mRNA expression levels of IL-1β, (b) relative mRNA expression levels of IL-6, and (c) relative mRNA expression levels of TGF-β; n = 6-8 in each group. Data are shown as individual plots and means ± SEM. ○: individual plot, *P<0.05. Data were analyzed using two-way ANOVA. RKT, rikkunshito; DW, distilled water; I/R, ischemia-reperfusion; IL-1β, interleukin-1β; IL-6, interleukin-6; TGF-β, transforming growth factor-β; SEM, standard error of means; ANOVA: analysis of variance

**Figure 6:**
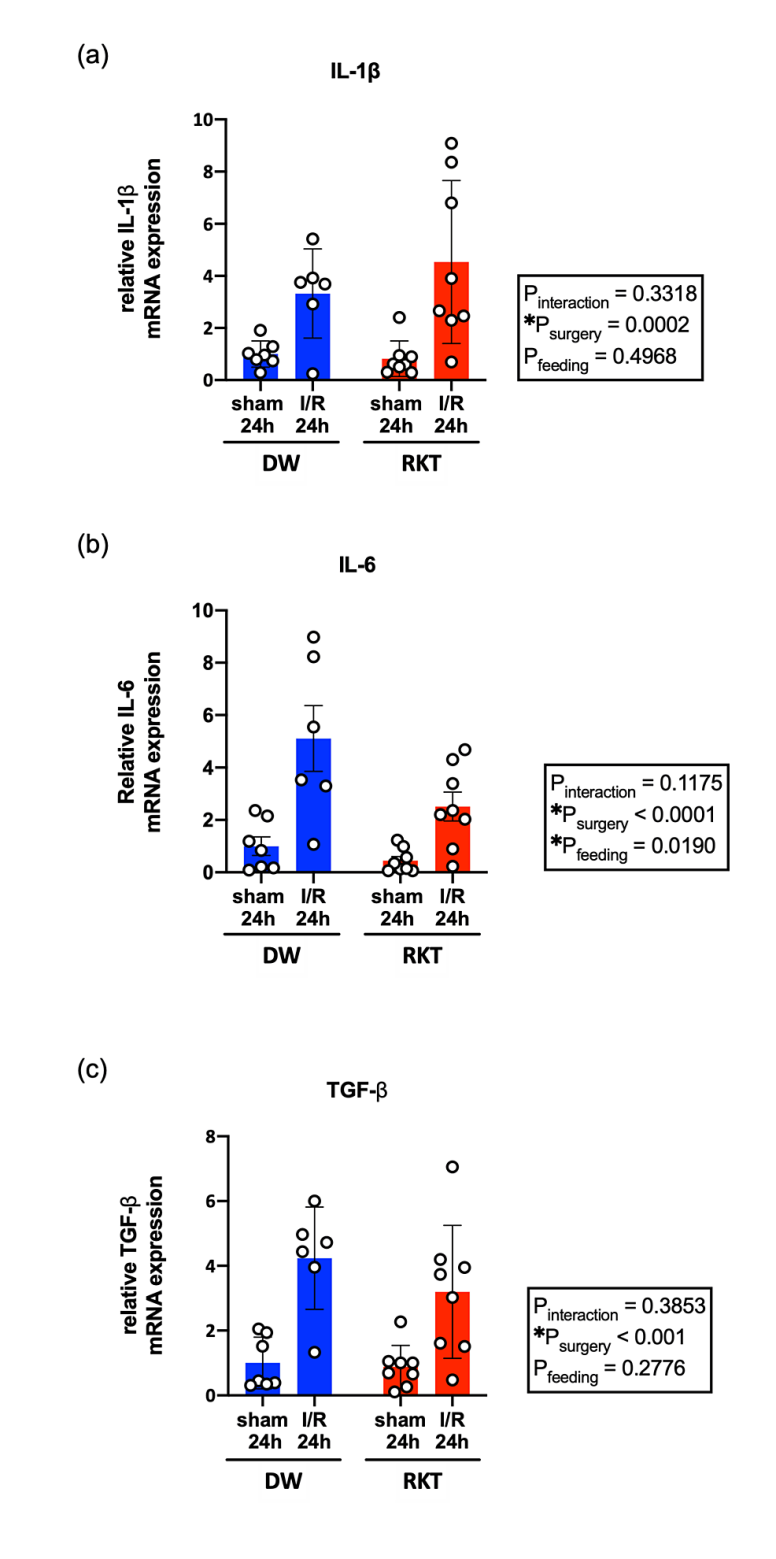
Myocardial mRNA expression levels of inflammation-related cytokines at 24 h of reperfusion (a) relative mRNA expression levels of IL-1β, (b) relative mRNA expression levels of IL-6, and (c) relative expression levels of TGF-β. n = 6-8 in each group. Data are shown as individual plots and means ± SEM. ○: individual plot, *P<0.05. Data were analyzed using two-way ANOVA. RKT, rikkunshito; DW, distilled water; I/R, ischemia-reperfusion; IL1-β, interleukin-1β; IL-6, interleukin-6; TGF-β, transforming growth factor-β; SEM: standard error of means; ANOVA, analysis of variance

To investigate the effect of RKT on cardiac inflammation induced by I/R injury in the acute phase, the cardiac gene expression levels of inflammation-related cytokines (IL-1β, IL-6, and TGF-β) were measured at 2 and 24 h of reperfusion (Figure 5 and Figure 6). I/R surgery had a significant effect on mRNA expression levels (P_surgery_ < 0.05), except for that of IL-6 in the I/R 2 h group (P_surgery_ = 0.1615, Figure 6). There was a statistically significant interaction in the mRNA expression levels of IL-1β at 2 h after reperfusion (P_interaction_ = 0.0475), and RKT significantly ameliorated the increase in mRNA levels of IL-1β in the RKT-I/R 2 h group compared to the DW-I/R 2 h group in multiple comparisons (P = 0.0387, Figure 5). Furthermore, the P values for the main effects of feeding (DW or RKT) were statistically significant in the analysis of TGF-β at 2 h of reperfusion (P_feeding _= 0.0045, Figure 5) and IL-6 at 24 h of reperfusion (P_feeding_ = 0.0190, Figure 6), indicating that RKT administration significantly reduced the basal expression levels of these genes in the heart.

Effect of RKT on the plasma concentration of ghrelin and expression of cardiac Sirt1 after I/R injury

**Figure 7:**
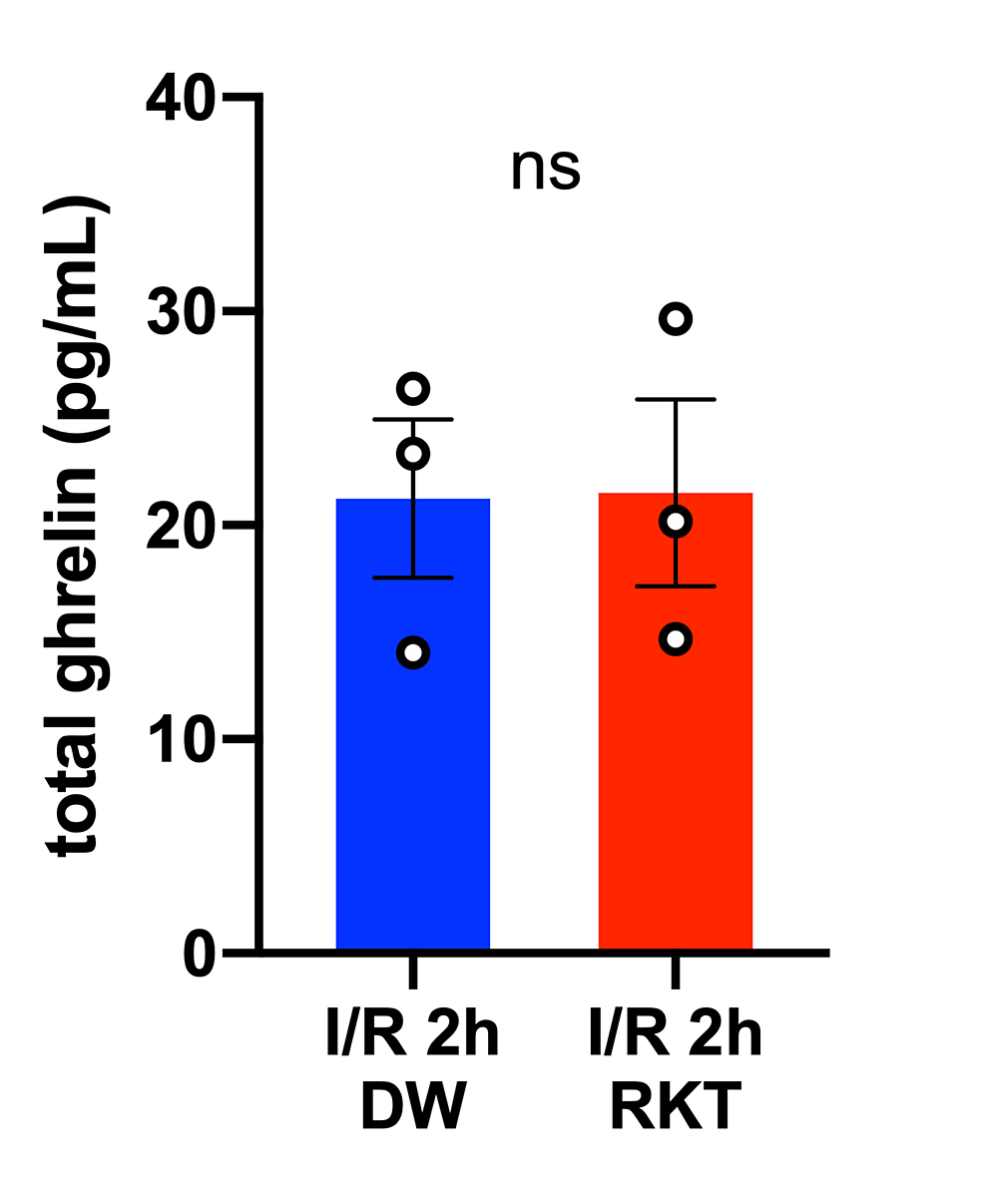
Total ghrelin concentration in plasma after 2 h of reperfusion n = 3 in each group. Data are shown as individual plots and means ± SEM. Data were analyzed using Student’s t-tests. ○: individual plot, ns: nonsignificant at P > 0.05. RKT, rikkunshito; DW, distilled water; I/R, ischemia-reperfusion; SEM, standard error of the mean

**Figure 8:**
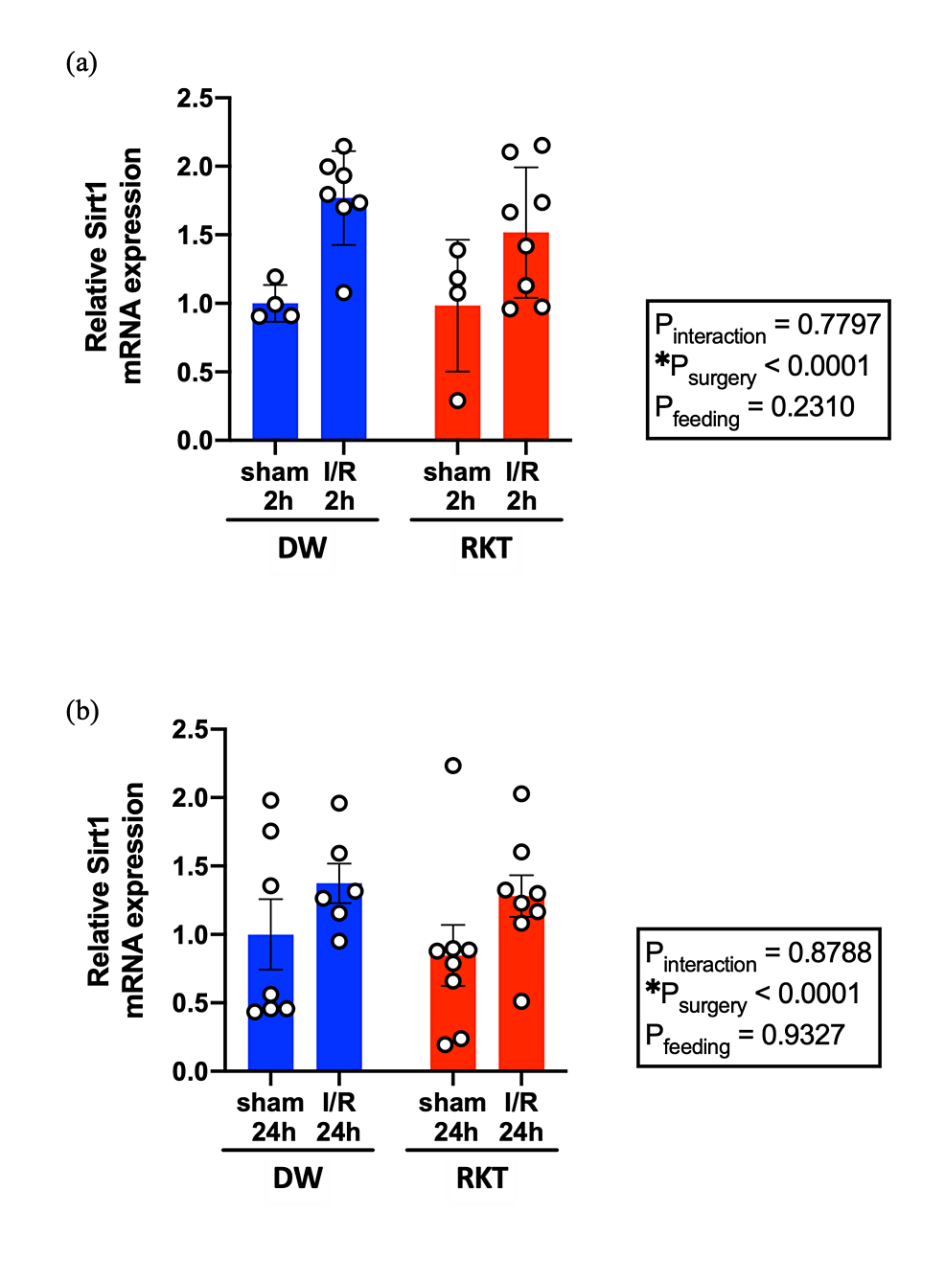
Myocardial mRNA expression levels of Sirt1 (a) After 2 h of reperfusion. (b) After 24 h of reperfusion. n = 4-8. Data are shown as individual plots and means ± SEM. ○: individual plot, *P<0.05. Data were analyzed using two-way ANOVA. RKT, rikkunshito; DW, distilled water; I/R, ischemia-reperfusion; Sirt1, sirtuin1; ANOVA, analysis of variance

Because RKT has been reported to stimulate ghrelin secretion, which is known to exert cardioprotective effects against I/R injury, plasma ghrelin levels were measured to assess the effect of RKT (Figure 7) [29,30,35-39]. Plasma ghrelin levels did not differ between the RKT and DW groups at 2 h after reperfusion (P = 0.0820). Additionally, because Sirt1 is an important downstream factor in the anti-inflammatory effect of RKT, the mRNA expression levels of cardiac Sirtuin 1 (Sirt1) were measured at 2 h and 24 h of reperfusion (Figure 8) [22,24]. Although the expression levels of cardiac Sirt1 were significantly upregulated by I/R surgery at 2 h and 24 h after reperfusion (P_surgery _< 0.0001), RKT did not show a significant effect on Sirt1 mRNA expression level (P_feeding_ > 0.05).

## Discussion

In the present study, pre-treatment with RKT for I/R injury showed partial suppression of some inflammatory cytokines in the acute phase after surgery but did not reduce myocardial damage. In the infarcted myocardium, an immune response occurs to reactive oxygen species production and myocyte death, activating and mobilizing neutrophils and monocytes, leading to an inflammatory response [6,40]. This reaction removes dead cells and extracellular debris from the infarct site, leading to myocardial healing. Proinflammatory cytokines such as TNF-α, IL-6, and IL-1β are upregulated markedly in experimental models of myocardial I/R injury in the acute phase [41,42]. Previous studies have reported that the suppression of these cytokines inhibits apoptosis and remodeling of cardiomyocytes [13,14,43,44]. In this study, as in previous ones, the mRNA expression levels of inflammation-related cytokines were increased following I/R injury [34,35]. In addition, pre-treatment with RKT suppressed the mRNA expression levels of cardiac IL-1β at 2 h after reperfusion and basal expression levels of cardiac TGF-β and IL-6. However, likely due to the limited anti-inflammatory effects of RKT, there was no significant reduction in myocardial IS. 

Fujitsuka et al. showed that oral administration of RKT for 100 days in a mouse model of accelerated aging significantly reduced cardiac calcification but did not affect ghrelin secretion [22]. Zhan et al. also reported that in a mouse model of angiotensin-induced atrial fibrillation for 14 days, simultaneous administration of RKT prevented atrial fibrosis and reduced inflammatory cytokine levels by enhancing the growth hormone secretagogue receptor-Sirt1 pathway, independent of ghrelin [24]. In the present study, consistent with previous studies [22,24], RKT did not stimulate ghrelin secretion. In the first study reporting that RKT stimulates ghrelin secretion, mice were administered RKT dissolved in water ad libitum. Differences in the methods of RKT administration may have affected ghrelin secretion. 

One reason that the anti-inflammatory effect of RKT was insufficient in this study may be related to the timing of RKT administration. Previous studies have reported the anti-inflammatory effects of RKT administered in combination with stress or injury [18,23,24,26]. Tsubouchi et al. investigated the anti-inflammatory effects of RKT in a bleomycin-induced model of acute lung injury [18]. According to the study, concomitant treatment with bleomycin reduced histological lung injury and inflammatory cytokines such as TNF-α, IL-1β, and IL-6, as well as the nuclear factor kappa-B. Interestingly, a reduction in lung injury was observed when RKT was administered post-treatment. As mentioned above, Zhan et al. administered RKT simultaneously with angiotensin II, and Fujitsuka et al. administered RKT for 100 days [24]. In the present study, pre-treatment with RKT suppressed some inflammatory cytokines but was not sufficient to reduce myocardial damage. Taken together, these results suggest that the anti-inflammatory effects of RKT may be exerted by administering RKT simultaneously during or after myocardial I/R injury post-treatment rather than pre-treatment.

Sirt1-mediated cardioprotective effects have been demonstrated in several studies [45-48]. For example, ischemic preconditioning for cardiac I/R injury has been reported to enhance Sirt1 expression [45]. Several studies have shown that the anti-inflammatory effects of RKT are mediated by growth hormone secretagogue receptor-mediated Sirt1 activation [22,24,26]. In the present study, Sirt1 mRNA expression was not altered by RKT pre-treatment. Wakui et al. reported that RKT did not suppress renal inflammation in the acute phase, and as they noted, Japanese herbal medicines have traditionally been used mainly for chronic diseases [26]. If RKT were administered continuously after I/R injury rather than before I/R injury, some beneficial effect on Sirt1 expression might have been observed.

The present study has some limitations. First, we only examined the effect of pre-treatment with RKT in the acute phase (first 24 h) after myocardial I/R injury. Further studies must clarify the effect of constant RKT administration after surgery on chronic conditions such as cardiac remodeling and fibrosis. Second, inflammation-related cytokines were measured only at two time points (2 h and 24 h). These time points were based on previous reports; however, other differences in the expression levels of cytokines might have been observed between the study groups at different time points [30,45,48]. Third, the effects of atractylodin, a component of RKT known to have a particular organ-protective effect, were not evaluated [15,24]. Because RKT contains eight kinds of herbal components, further research with specific herbal medicines, such as atractylodin, may reveal a component that is more effective for myocardial I/R injury.

## Conclusions

In a mouse model of in vivo myocardial I/R injury, two week pre-treatment with RKT did not reduce myocardial damage in the acute phase but did suppress some inflammation-related cytokines. Because inflammation-related cytokines are involved not only in myocardial damage in the acute phase of I/R injury but also in the repair process in the chronic phase, it is promising that long-term postoperative administration of RKT may have some beneficial effects.
